# A Cycling Movement Based System for Real-Time Muscle Fatigue and Cardiac Stress Monitoring and Analysis

**DOI:** 10.1371/journal.pone.0130798

**Published:** 2015-06-26

**Authors:** Szi-Wen Chen, Jiunn-Woei Liaw, Ya-Ju Chang, Hsiao-Lung Chan, Li-Yu Chiu

**Affiliations:** 1 Department of Electronic Engineering, Chang Gung University, Tao-Yuan, Taiwan; 2 Heathy Aging Research Center (HARC), Chang Gung University, Tao-Yuan, Taiwan; 3 Department of Mechanical Engineering, Chang Gung University, Taoyuan, Taiwan; 4 Department of Physical Therapy and the Graduate Institute of Rehabilitation Science, College of Medicine, Chang Gung University, Taoyuan, Taiwan; 5 Department of Electrical Engineering, Chang Gung University, Taoyuan, Taiwan; University of Miami School of Medicine, UNITED STATES

## Abstract

In this study, we defined a new parameter, referred to as the cardiac stress index (CSI), using a nonlinear detrended fluctuation analysis (DFA) of heart rate (HR). Our study aimed to incorporate the CSI into a cycling based fatigue monitoring system developed in our previous work so the muscle fatigue and cardiac stress can be both continuously and quantitatively assessed for subjects undergoing the cycling exercise. By collecting electrocardiogram (ECG) signals, the DFA scaling exponent α was evaluated on the RR time series extracted from a windowed ECG segment. We then obtained the running estimate of α by shifting a one-minute window by a step of 20 seconds so the CSI, defined as the percentage of all the less-than-one α values, can be synchronously updated every 20 seconds. Since the rating of perceived exertion (RPE) scale is considered as a convenient index which is commonly used to monitor subjective perceived exercise intensity, we then related the Borg RPE scale value to the CSI in order to investigate and quantitatively characterize the relationship between exercise-induced fatigue and cardiac stress. Twenty-two young healthy participants were recruited in our study. Each participant was asked to maintain a fixed pedaling speed at a constant load during the cycling exercise. Experimental results showed that a decrease in DFA scaling exponent α or an increase in CSI was observed during the exercise. In addition, the Borg RPE scale and CSI were positively correlated, suggesting that the factors due to cardiac stress might also contribute to fatigue state during physical exercise. Since the CSI can effectively quantify the cardiac stress status during physical exercise, our system may be used in sports medicine, or used by cardiologists who carried out stress tests for monitoring heart condition in patients with heart diseases.

## Introduction

Fatigue is often considered as an important indicator in diseases due to abnormality or a progressive decline in motor function during motor tasks [1–7]. In fact, on-line fatigue detection and monitoring is important over many aspects of interventions in physical therapy or exercise training. For example, without knowing the relative levels of fatigue and onset of fatigue, it would be very difficult to design effective training programs for patients and athletes. Due to the convenient, safe, and effective cardiovascular exercise it may provide, a stationary bicycle, or alternatively called an exercise bicycle, has been typically used as physical therapy or exercise training equipment. Cycling-based movement, by definition, is to perform rhythmic movement trainings on a stationary bicycle. In general, cycling-based movement can provide a low-impact, safe and effective way for walking training as well as the lower limb coordination training [8]. Previous researches in literature have also indicated that cycling exercise can provide a convenient paradigm used in patients with cardiac disease [9], or neurological disorder [10]–[11], and in athletes [12]. Although cycling exercise can effectively induce fatigue, there still remains a deficiency in developing useful quantification methods for on-line monitoring and analysis of fatigue during exercise.

In fact, fatigue can be classified as central and peripheral fatigue; the former is associated with the central nervous system (CNS) while the latter is associated with the peripheral neuromuscular system [13]–[14]. The rating of perceived exertion (RPE) scale is considered as a convenient index which is commonly used to monitor subjective perceived exercise intensity. In general, RPE would rise as fatigue rises during exercise, and hence one can determine the level of fatigue simply by monitoring this rise in RPE. For example, there is a previous study showing that, during fatigue induced by stepping exercise, the increase in RPE was related to both the cardiovascular status reflected by heart rate, and the local muscle factors monitored by the median frequency of electromyogram (EMG) [14]. In addition, Stamford indicated that a linear relationship might exist between RPE and HR during progressively increasing workloads and submaximal constant load [15]. Borg also suggested that there exists a high correlation between ten times the value of RPE and actual HR during physical exercise and this actually gives a good estimate of actual HR during exercise. However, due to its subjectivity and incompleteness, RPE may not be optimal to quantify fatigue during exercise. That is, fatigue is a more complex phenomenon that may not be solely related to nor reflected by RPE. Therefore, development of simple, reliable and objective indices, other than RPE, based on the local muscle factors (i.e., the motor-related factors) and the factors that influence HR dynamics (i.e., the non-motor-related factors) for monitoring fatigue state during exercise is demanded.

Considering the local muscle factors first, a reduction in median frequency of the EMG power spectrum has been widely accepted as an indicator of fatigue since it has been observed in fatigue induced by maximum and sub-maximum voluntary contractions [16–19]. In fact, such a shift in the power spectrum can be attributed to motor units recruitment [17], [20], fatigue-induced metabolic accumulation [21], change in intracellular PH [22], and reduction in muscle fiber conduction velocity [16], [23–25]. It is worth noting that we have developed a cycling based fatigue monitoring and analysis system for continuously and quantitatively evaluating the progression of fatigue in a previous work [26]. In that study, we have proposed a novel time-varying parameter, called the fatigue progression measure (FPM), to quantify the local muscle fatigue so the onset of the occurrence of fatigue can be explicitly determined from the FPM tracings, which was considered unprecedented.

However, one of the interesting questions usually arising from endurance training is often asked: “Is the one who underwent such a training test truly fatigued or just lacking in motivation?” Seeking for the answers to the question actually motivated this study. Although heart rate (HR) would increase when one exercises, an increase in HR does not necessarily indicate fatigue. On the other hand, fatigue induced by physical exercise may be partly attributed to an increase in cardiac stress. In fact, the idea of a relationship between cardiac stress and fatigue is not original. Since intensive exercise will be obviously related to incremented sympathetic tone and reduced parasympathetic tone, heart rate variability (HRV) analysis may provide a more effective and reliable way for figuring out this relationship. In this aspect, some previous researches in literature, for instance, have reported that HRV may and does show influence of mental and physical stress [27]–[28].

It has been widely accepted that the autonomic nervous system (ANS) plays a direct role in the ruling action of the HR control. The CNS in brain receives various stimuli and responds to these stimuli via the ANS activity. The ANS activity modulates the heart rate as well as other cardiovascular variables, such as blood pressure, and then feeds back to the CNS again. Since such a control is close-loop and running continuously, the HRV analysis thus provides a noninvasive way for the study of ANS modulation. There are a number of previous studies in the literature having indicated that HRV analysis may provide an insight into the mechanism of ANS activity [29–31]. In general, HRV is defined by quantifying the variation in heart beats or interbeat intervals, the so called RR intervals, based on either the time- or frequency-domain analysis. In addition, some researchers also employed a fractal measure, called the detrended fluctuation analysis (DFA), to quantitatively characterize the HR dynamics, and suggested that the DFA might be well suited for non-stationary situations, such as incremental exercise [32]–[33]. In fact, it has been accepted that fractal-like characteristics of HR can be considered as an indication of a normal and healthy heart since fractal measure of HR has manifested itself to change towards uncorrelated randomness for those who were suffering from different heart diseases or in the aging population [33–35].

As mentioned in the very beginning, we have developed a cycling movement based system for on-line fatigue monitoring and analysis in a previous work. In this study, we further examined the effects of cycling exercise on HR dynamics at a constant load. The hypothesis of our study was partly based on an extension of another previous work in literature regarding the DFA with its applications into heart rate dynamics on aerobic cycling exercise tests [33]. Although that work has successfully enabled the applications of DFA scaling exponent into cycling exercise with attractive features, particularly a decrease in *α* during the graded cycling exercise test, all their data processes were done after all the RR data were collected, i.e. not real-time. In this study, we defined an innovative time-varying parameter based on the DFA scaling exponent, dubbed the cardiac stress index (CSI), and incorporated it into the cycling based fatigue monitoring system developed by us previously so the muscle fatigue and cardiac stress can be synchronously monitored during the physical exercise. We believe such a novel system can be used in sports medicine, or used by cardiac clinicians for on-line monitoring heart condition in patients with heart diseases when undergoing stress tests.

## Methods and Materials

The study protocol had been approved by Chang Gung Medical Foundation Institutional Review Board (IRB no.101-5141B) in accordance with the Helsinki Declaration. All participants have provided their written consent, approved by Chang Gung Medical Foundation Institutional Review Board (IRB no.101-5141B), to participate in this study.

### An overview of our cycling-based ergometer system

**Fig 1:**
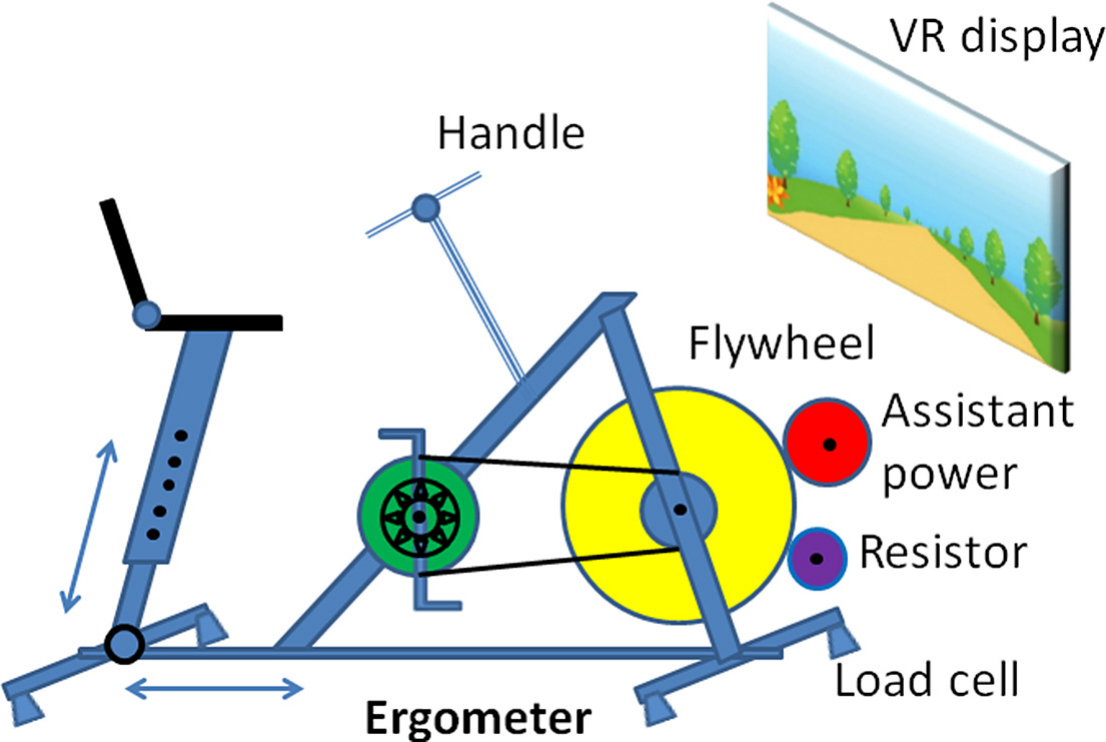
The schematic diagram of the ergometer used in our cycling-based real-time fatigue and cardiac stress monitoring and analysis system. It mainly consists of a stationary bicycle equipped with a resistor, crank angle detector and the wireless EMG and ECG sensors with sensor interface devices.

In order to perform rhythmic movement trainings, a stationary bicycle based ergometer system was developed and used. The information regarding our cycling-based ergometer system is as depicted in Fig 1 and described in detail previously [26]. In addition, in this study the system further equipped with a set of wireless ECG sensors so the ECG signals can be collected and then processed by the system PC in a real-time manner.

**Fig 2:**
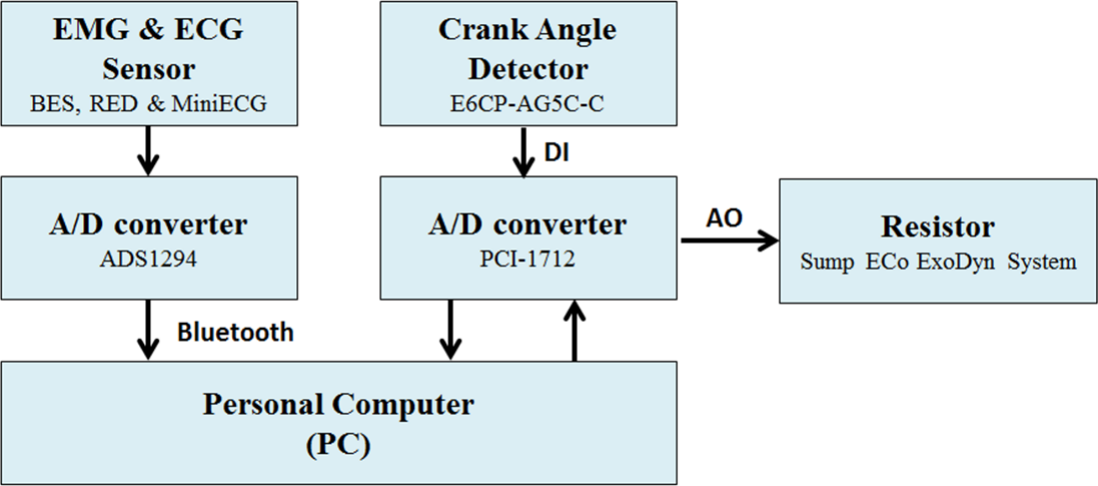
Schematic block diagram of overall system configuration.

Fig 2 shows the schematic block diagram of the overall system configuration. According to the schematic block diagram as depicted in Fig 2, we here use an optical encoder (or alternatively known as the crank angle detector) to estimate the rotation speed of the cycling movement on a real-time basis. Moreover, a resistor-based load control device is incorporated into our system to provide designated amount of workload imposed to the cycling-based training.

We here adopted EMG and ECG sensors to collect medical data from the subjects. The software programs were devised to process these medical signals to extract the diagnostically useful information in an integrated fashion. In summary, the system PC was used to carry out the on-line analyses as follows: 1) acquiring and displaying the ECG, EMG, HR, and cycling speed data, 2) preprocessing the collected raw ECG and EMG data, 3) computing the fast Fourier transform (FFT) based power spectrum and the DFA scaling exponent, 4) transforming all the numerical results into fatigue and cardiac stress related parameters (*e*.*g*., FPM and CSI) used for on-line fatigue and cardiac stress monitoring and analysis.

When cycling started, the wearable wireless sensors synchronously collected both EMG and ECG data from the subject and then transmitted them to an A/D converter so the system PC may acquire the digital data for on-line monitoring and analysis during the exercise. In addition, other measures of interest such as the mean cycling speed and instantaneous heart rate were also on-line monitored so a visual feedback can be provided for the subject while undergoing the test.

### Participants and experimental procedure

There were twenty-two healthy adults recruited for undergoing the bipedal cycling test in this study. All the participants were healthy and free of any muscular or cardiac diseases. Their mean age was 22.8 years old and they all had at least 10 years cycling experience. The procedures of the experiment and the characteristics of the bicycle ergometer system were clearly explained to them before proceeding. Each participant was asked to do one-minute warm-up exercise to prevent sports injuries. Then, the experiment started and each participant was asked to sit in an upright position and perform cycling exercise under a constant load throughout the entire course of experiment with a constant speed of 60 RPM. During the cycling process, EMG of vastus lateralis (VL) and gastrocnemius (GAS) muscles, ECG, instantaneous HR, cycling velocity and cycling time were all synchronously recorded.

In addition, it has been known that the Borg rating of perceived exertion (RPE) scale is used to reflect how hard a participant feels about the work when exercising [36]. With ranging from 6 to 20, the Borg scale value was also orally rated by each participant every minute during the experiment. For each participant, the experiment was terminated immediately once his Borg scale value reached the maximal value of 20.

### ECG preprocessing settings and detrended fluctuation analysis (DFA) on HR dynamics

In addition to the EMG-based fatigue analysis, in this study the detrended fluctuation analysis (DFA) was further introduced to the characterization of HR dynamics. For this purpose, the subject’s surface ECG was measured and digitized by an ECG sensor interfacing system first. The digital ECG data were then wirelessly transmitted to the system PC through Bluetooth. Note that here the sampling rate was set to 200Hz and the raw ECG data was preprocessed by a bandpass filter. After detecting the time locations of the R waves for an ECG signal using a template-match technique that involves a correlation coefficient based similarity calculation, we may obtain the interbeat interval (i.e., the RR interval) data sequence corresponding to that ECG. On the other hand, it should be also noted that for online or mobile HRV analysis ectopic beats would generally cause abnormal RR intervals, or alternatively known as the outliers, that occur randomly and are longer or shorter than normal RR data. This would represent a major source of error when analyzing HRV data in both the time and frequency domain. Therefore, in order to solve this problem, an algorithm used for detecting and editing RR outliers was applied before performing DFA. In the editing process, we deleted the RR outliers detected and then replaced the deleted ones simply by linearly interpolated RR values. In fact, some previous researches in literature reported that the effects of editing in DFA calculation may not be significant when the number of outliers is small (typically 5–10%) [37–39]. In our study, the DFA scaling exponent was thus evaluated on the edited RR time series.

DFA, first proposed by Peng *et al*. [32], is an algorithm that has proven useful in measuring the long-term autocorrelation of non-stationary time series. It can quantitatively characterize the complexity of time series using the fractal theory [32], [40]. In fact, previous researches in literature have indicated that the DFA scaling exponent, or alternatively known as *α*, can be used for classifying biomedical signals into the healthy and unhealthy classes [41]. In general, DFA simply analyzes the mean square deviation of a signal from its local trend line on a variety of scales. In short, taking a time series of interest, denoted as *x*(*i*), first an integrated time series, denoted as *y*(*k*), can be expressed as

$$y(k)=\sum_{i=1}^{k}\left[x(i)-\bar{x}\right]$$ (1)

where $\bar{x}$ represents the mean of *x*(*i*). Next, we divided *y*(*k*) into a number of equal-length segments. For each segment of length *n*, a least squares line is fit to the data. Note that the least squares line is thought of as being the “trend” in that segment. Then, we detrend the integrated time series *y*(*k*) by subtracting the local trend (i.e., the *y* coordinate of the least squares line segments), denoted as *y*_*n*_(*k*), from each segment separately. The root-mean-square based fluctuation, denoted as *F*(*n*), of this detrended time series is then calculated by

$$F(n)=\sqrt{\frac{1}{N}\sum_{k=1}^{N}{\left[y(k)-y_{n}(k)\right]}^{2}}$$ (2)

In order to characterize the relationship between *F*(*n*) and the time scale *n* (i.e., segmental length), the calculation in (2) is repeated until all possible values of *n* are applied. In general, a linear relationship is seen on a log-log plot and the fluctuations can be then characterized by the slope of the line relating log *F(n)* to log *n*. Also note that in our study, *n* ranged from 4 to 64.

### An innovative new-defined indicator of cardiac stress–CSI

In fact, DFA can be used to quantify fractal-like autocorrelation properties of the biomedical signals which are usually considered complex and time-varying. For example, it has been applied to investigations into studies of HR dynamics and difference was actually seen between the values of scaling exponent *α* derived from healthy and unhealthy (i.e., due to diseases or aging) subjects, respectively [40–43]. In general, DFA scaling exponent *α* derived from healthy subjects appeared larger than that derived from unhealthy ones, implying that the HR time series corresponding to the unhealthy might be less regular and/or more complex than those corresponding to the healthy [33]–[35]. Moreover, it is also indicated by previous researches in literature that the DFA scaling exponent *α* might be well suited for studying HR dynamics under the situation like incremental exercise and could provide as an indication of cardiac stress status [32]–[33]. Therefore, we simply employed *α* to devise an innovative time-varying parameter, dubbed cardiac stress index (CSI), for continuously, quantitatively characterizing the cardiac stress status. The CSI is defined as

$$CSI=\frac{\text{Number of events with}\;\alpha \;\text{lower than}\;1}{\text{Total number of events}}$$ (3)

Similar to the way that FPM is defined (as described in detail previously [26]), (3) is a time function which can provide the running estimates of the percentage of less-than-one *α* counts. After cycling exercise starts, the denominator in (3) will be incremented by one every time when there is a new *α* value produced, while the numerator is incremented by one only when *α* is lower than 1. According to the definition in (3), the CSI can be used as a diagnostically useful indicator to continuously and quantitatively describe the progression of cardiac stress status as time evolves.

Recall in our previous study, we have devised another time-varying parameter, dubbed fatigue progression measure (FPM), based on the percentage of reduced median frequencies (MFs) of the EMG spectrum for quantitatively and continuously measuring the degree of fatigue [26]. According to [26], the FPM is defined as

$$FPM=\frac{\text{Number of events with MF lower than the reference}}{\text{Total number of events}}$$ (4)

**Fig 3:**
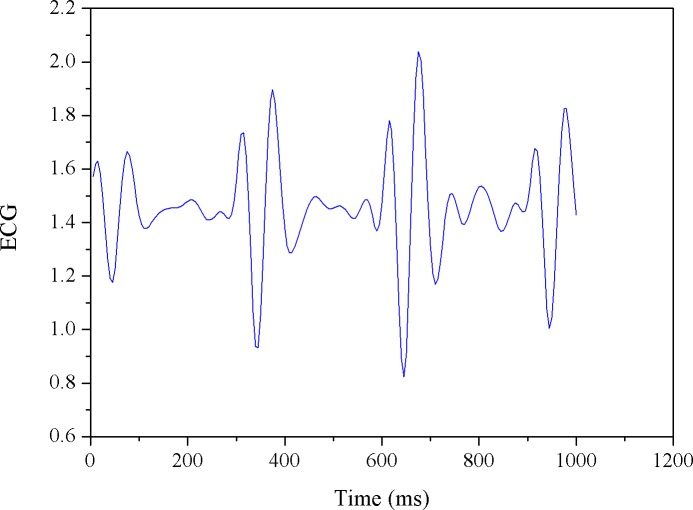
A preprocessed ECG segment measured during a cycling test.

**Fig 4:**
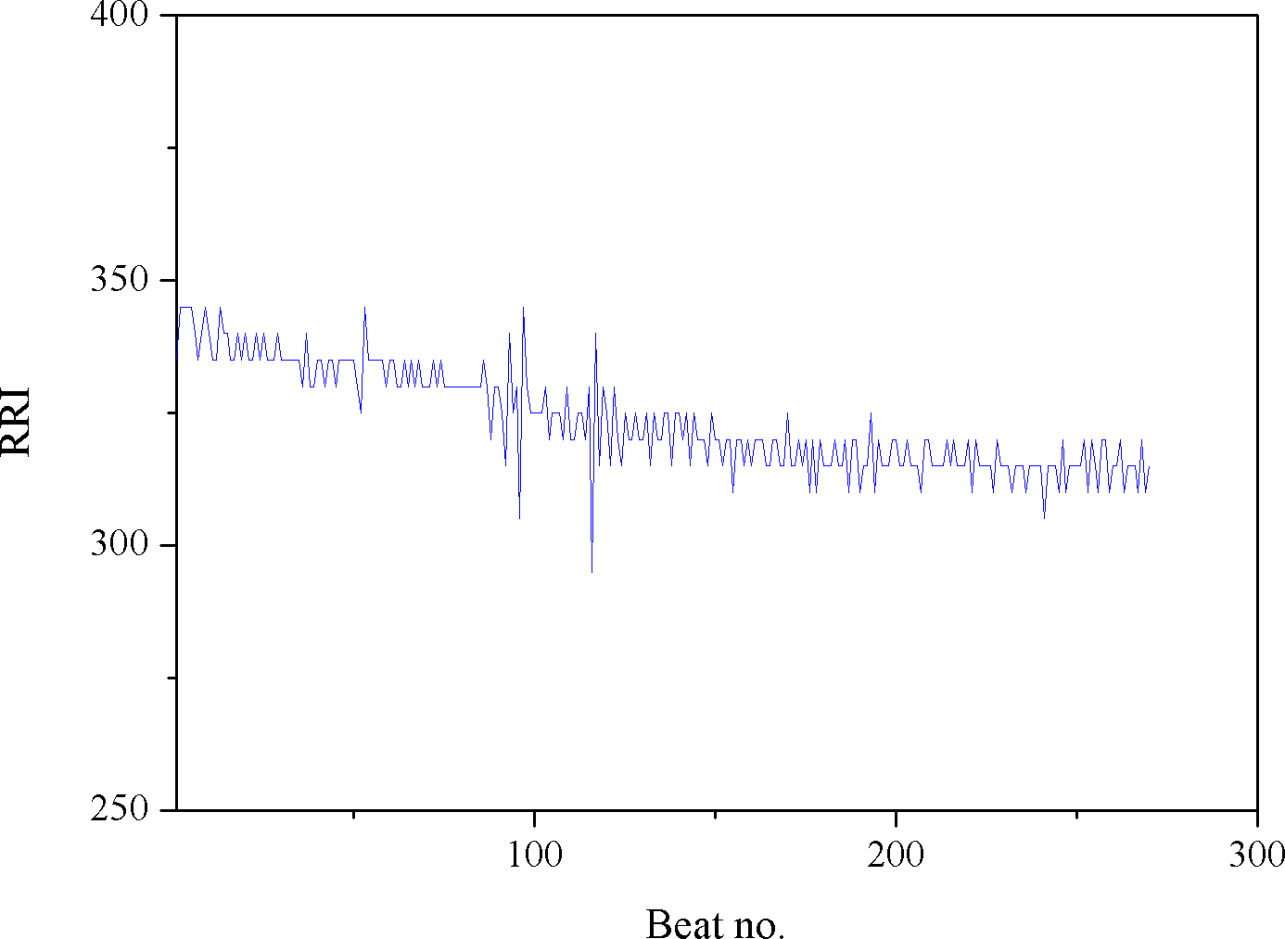
An RR data segment.

**Fig 5:**
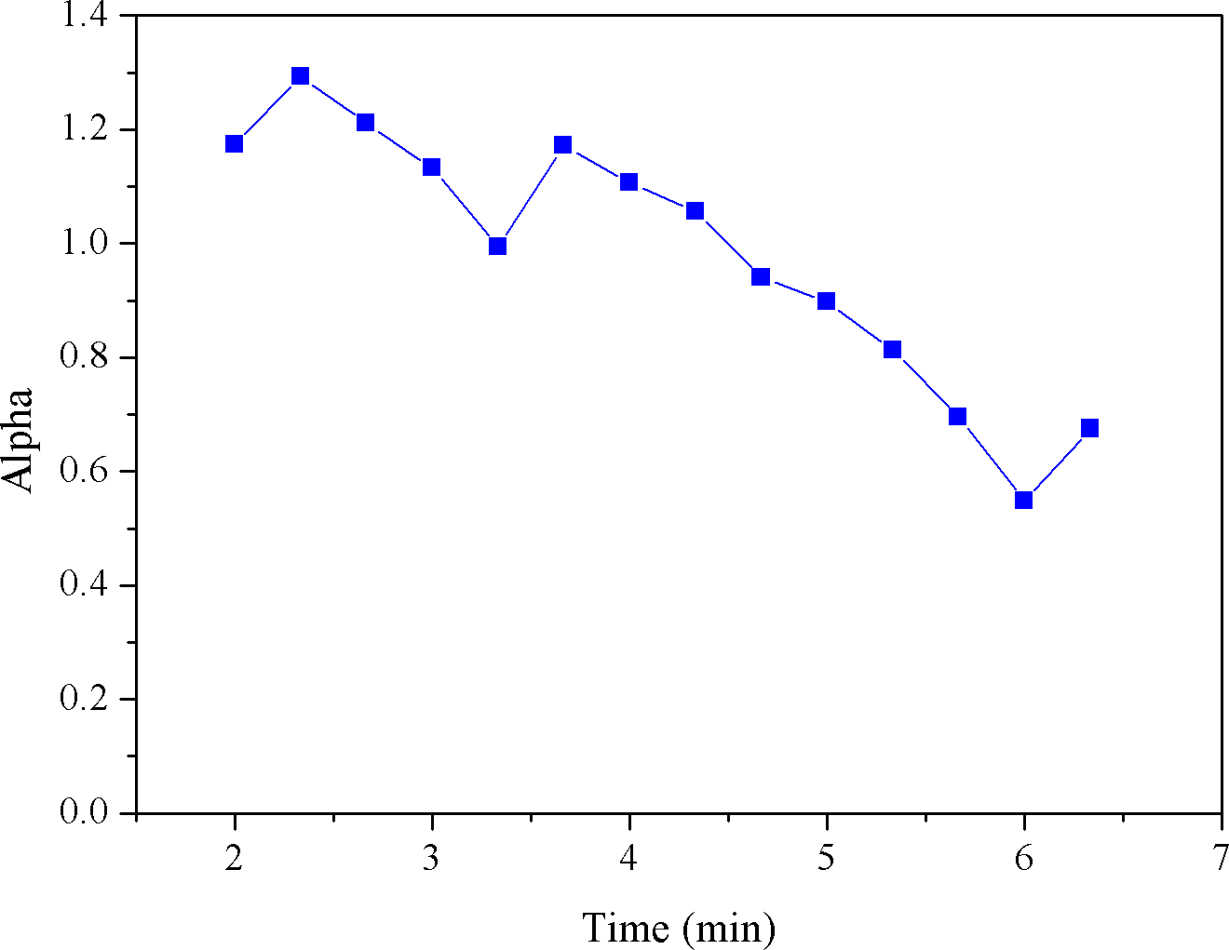
DFA scaling exponent *α* estimates derived from the RR time series.

**Fig 6:**
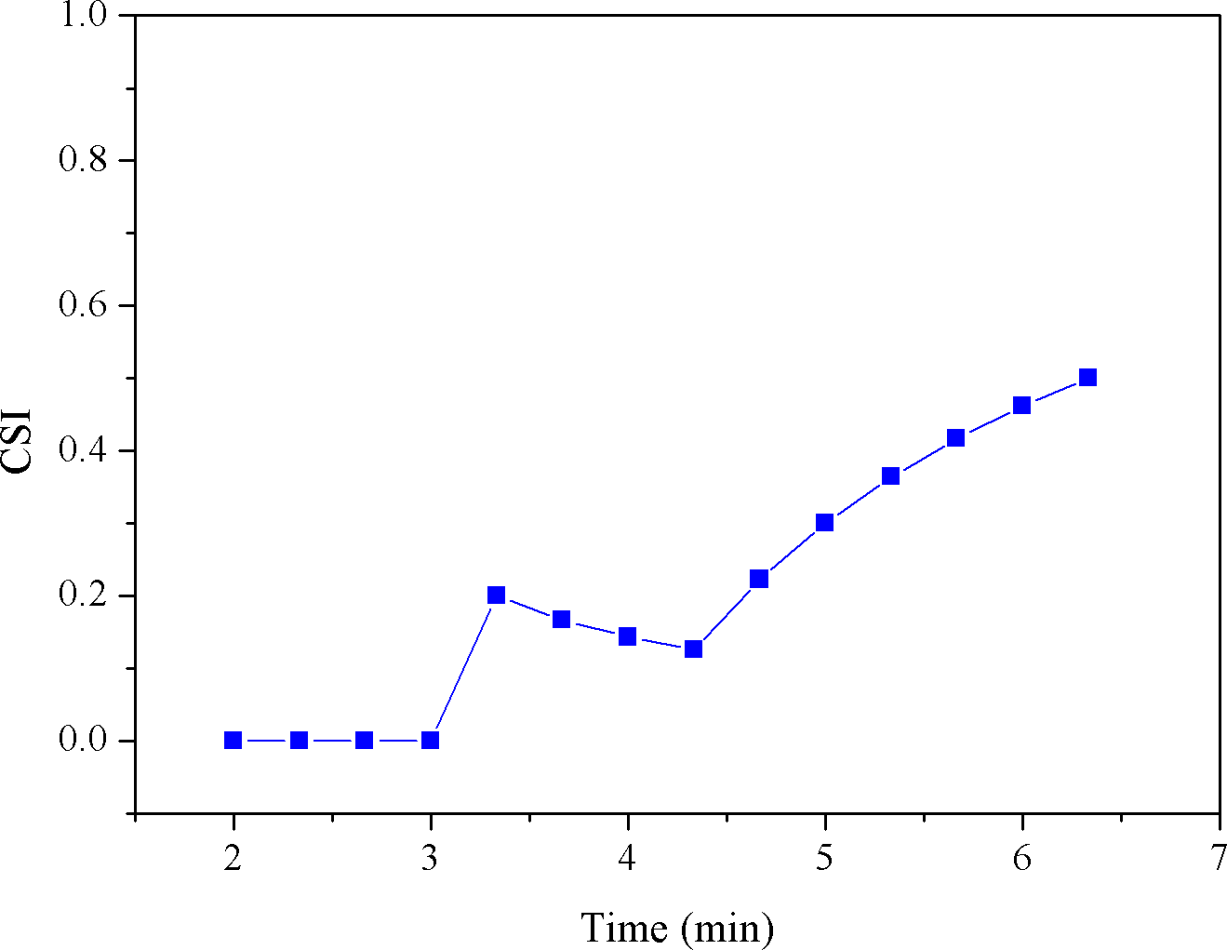
CSI tracings.

**Fig 7:**
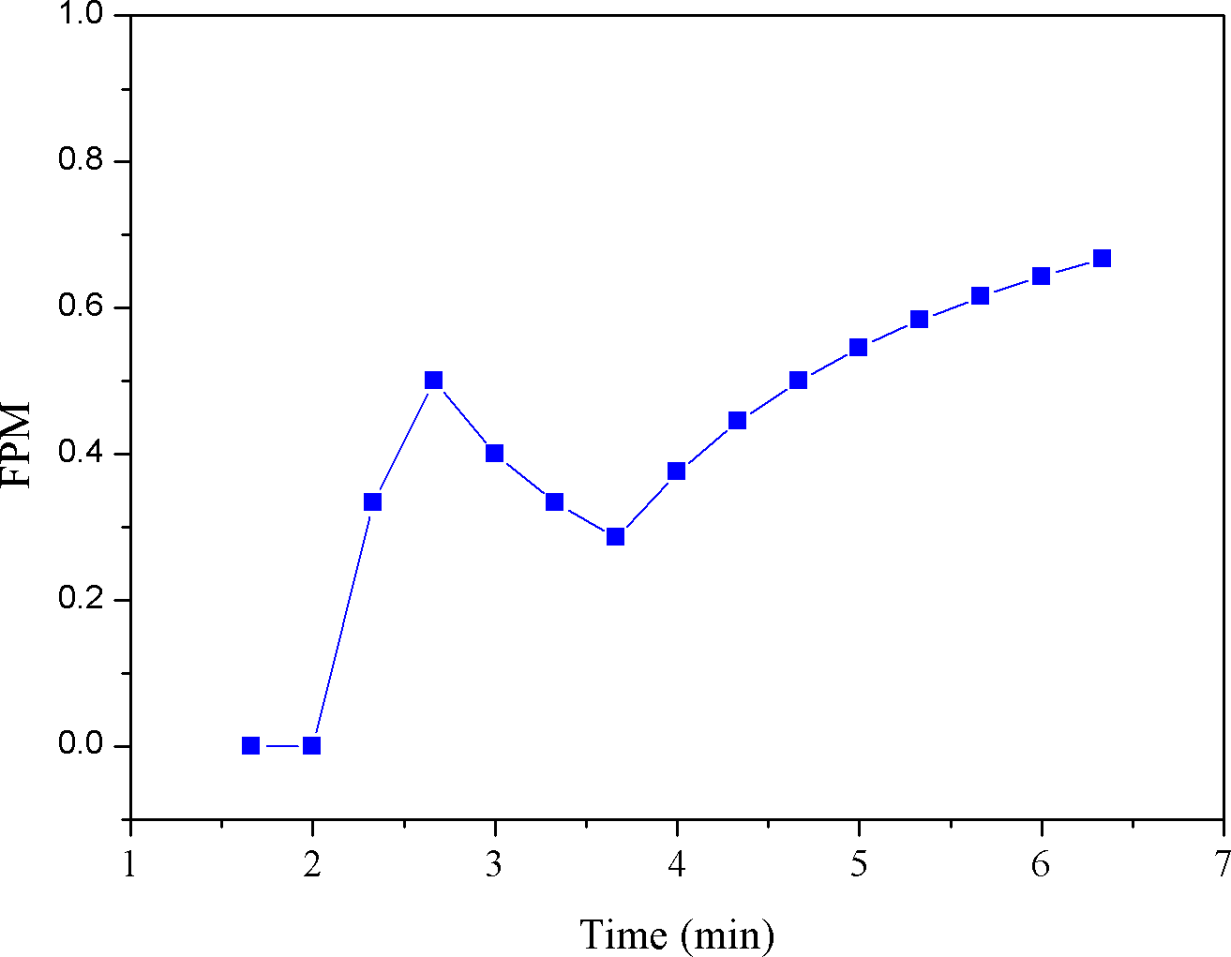
FPM tracings.

Here, we simply set the reference (i.e., the threshold) as first MF value obtained from the cycling test. Note that since MF is updated as time evolves, the FPM (i.e., the percentage of reduced MFs) would be synchronously updated. For example, given 10 MF numbers, when the threshold is crossed at the 2^nd^ number, the FPM sequence would be 0/1, 1/2, 2/3, 3/4, 4/5,…, 9/10; if crossed at the 3^rd^ point, it would be 0/1, 0/2, 1/3, 2/4, 3/5, 4/6, … 8/10. Obviously, FPM value will remain at zero until there is a MF number crossing (i.e., smaller than) the threshold. In fact, in our latest system, as presented in this study, the *α* value and the filtered MF value were both synchronously produced every 20 seconds and thus, the time-varying parameters, FPM and CSI, were updated every 20 seconds to provide the continuous measures of fatigue and cardiac stress status, respectively. Figs 3–7 provide a typical real-time profile obtained during a cycling test, consisting of a bandpass filtered ECG signal, an RR data segment, DFA scaling exponent *α* tracings, CSI and FPM tracings, respectively.

Fig 3 shows a 1-second bandpass filtered ECG segment measured during the cycling test. The corresponding RR interval data sequence derived from the ECG signal was then obtained, as shown in Fig 4. Next, the running estimate of DFA scaling exponent *α* throughout the entire cycling test can be derived, as depicted in Fig 5. Here, we simply evaluated *α* (*t*_*k*_) by shifting a 1-minute analysis window centered at *t*_*k*_ by a step of 20 seconds on the RR data. Observing Fig 5, a decrease in *α* was seen. This might be regarded as the result of an increase in cardiac stress [33]. The CSI tracings corresponding to the *α* signal were further evaluated and updated by successively comparing the *α* values with the reference value, 1.0, using (3) and plotted in Fig 6. Inspecting Fig 6, we may see that just like the FPM, the CSI can be used to assess the cardiac stress progression as well as detect the onset time at which the cardiac endurance starts to decrease. Fig 7 shows the real-time FPM estimates derived from the VL muscle measured during the test.

## Results and Discussion

As mentioned previously, we collected the raw EMG as well as ECG data from twenty-two subjects during the cycling tests in order to evaluate the overall performance of our system with its applications into real-time muscle fatigue and cardiac stress assessment. Note that among all the physiological measures employed in our system, the CSI method as described in previous subsection is innovative and new. Being similar to the FPM with respect to continuous muscle fatigue monitoring, the CSI can not only detect the onset time at which the cardiac endurance starts to decrease, but also continuously measure the progression of cardiac stress. Therefore, we believe this would represent the most significant benefit from this work. The subsequent numerical analysis results and discussions will be focused on the performance evaluation of both the FPM and CSI methods.

### Results of parameter trend analysis

**Fig 8:**
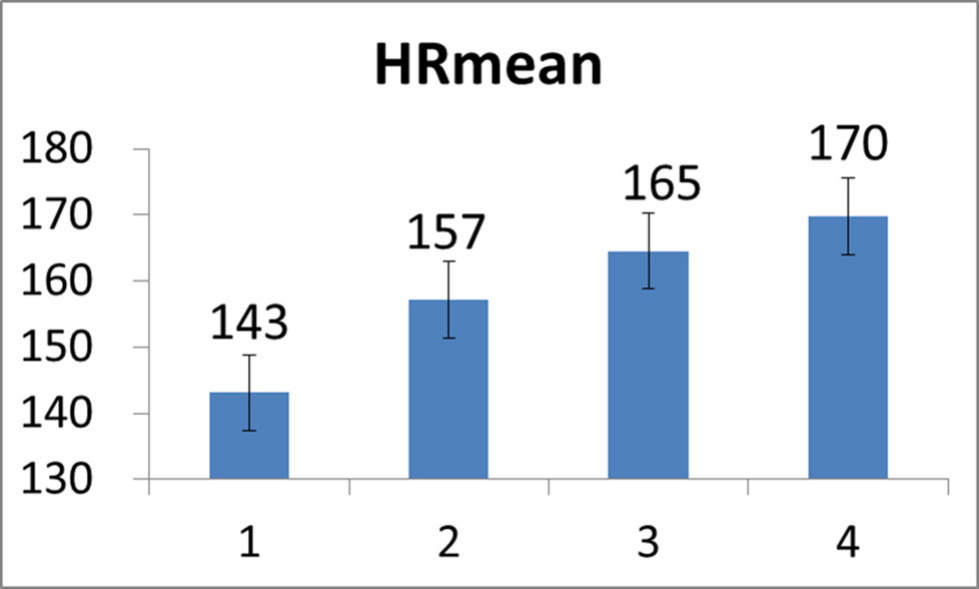
Mean HR values evaluated over all the subjects at the four stages of the cycling movement.

**Fig 9:**
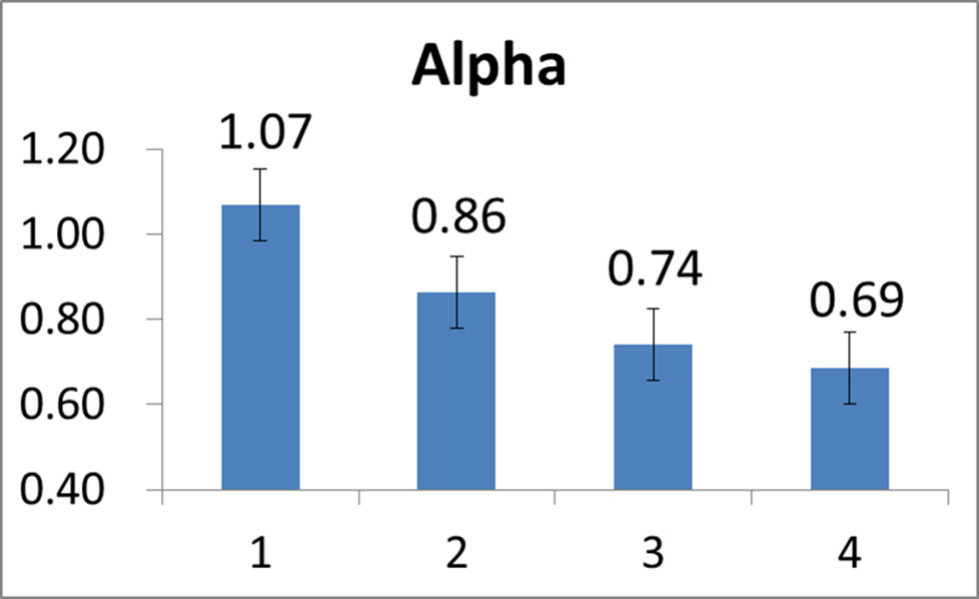
Mean *α* values evaluated over all the subjects at the four stages of the cycling movement.

**Fig 10:**
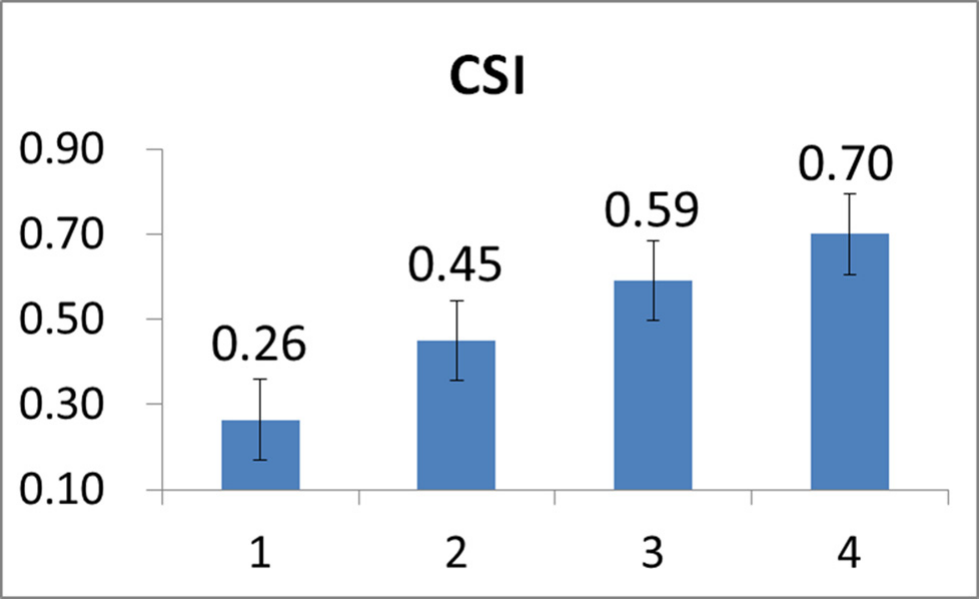
Mean CSI values evaluated over all the subjects at the four stages of the cycling movement.

Note that in our study, a constant load was applied for all the twenty-two subjects. Evaluation on CSI stopped at the time when the subject’s Borg RPE scale value reached 20. First, it should be noted that since there was considerable diversity in the cycling time of all the subjects undergoing the tests, in order to facilitate the task of performance evaluation we here evenly divided the cycling time interval of each subject into four sections/stages so the stochastic analysis may be conveniently performed. Figs 8–10 depict the mean HR, mean *α*, and mean CSI, respectively, of all the twenty-two subjects evaluated at the four stages in the course of cycling exercise. It is revealed from these figures that while the mean HR of these subjects increased from 143 beats/min to 170 beats/min during the cycling exercise, the mean DFA scaling exponent measure *α* decreased from 1.07 to 0.69, implying that the HR became less regular and/or more complex as the cycling time increased and thus the CSI increased from 0.26 to 0.70 (the maximum value of CSI is 1.0), as expected.

**Fig 11:**
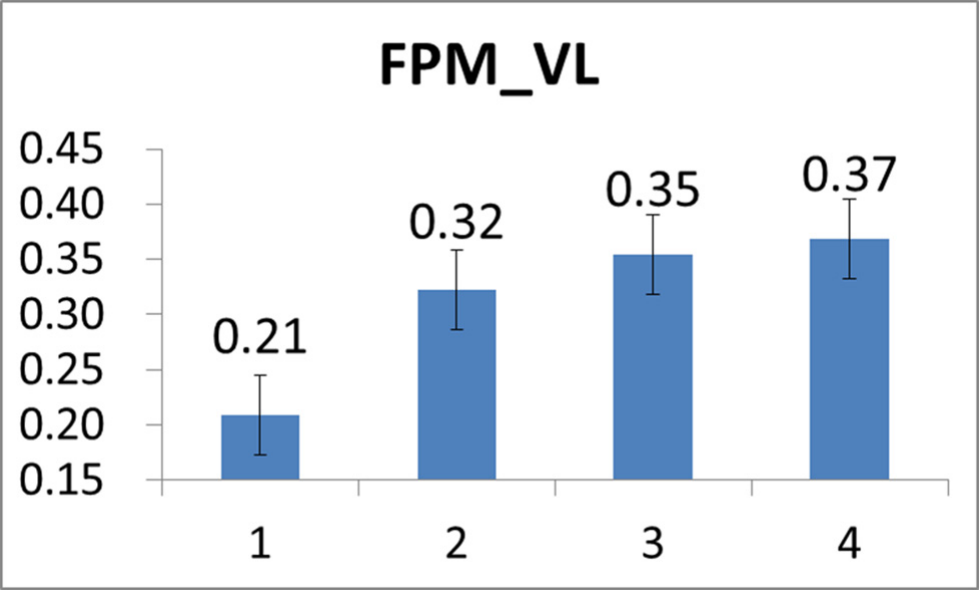
Mean FPM values of VL evaluated over all the subjects at the four stages of the cycling movement.

**Fig 12:**
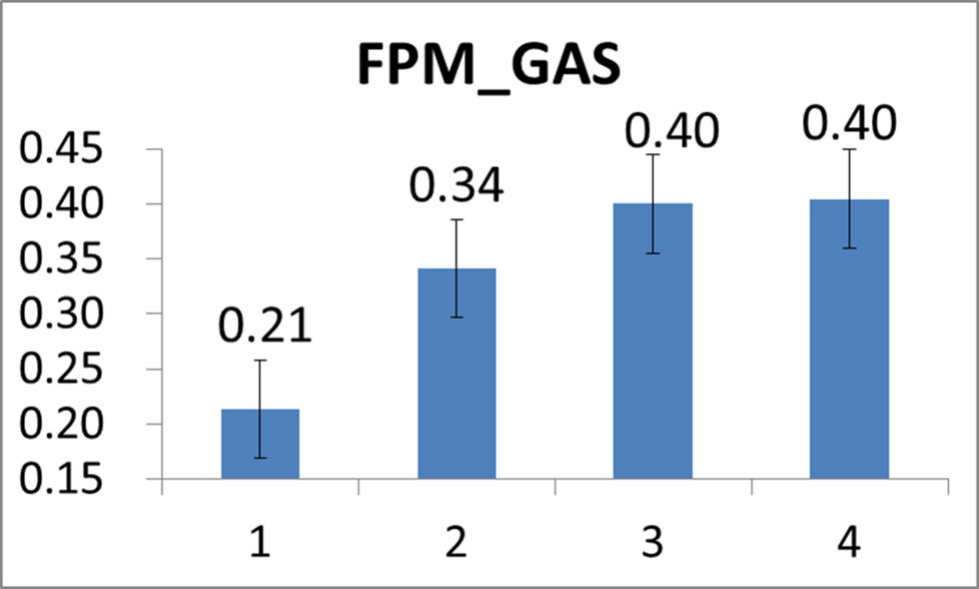
Mean FPM values of GAS evaluated over all the subjects at the four stages of the cycling movement.

Moreover, we also depicted the mean FPM plots derived from the twenty-two subjects’ VL and GAS muscles in Figs 11 and 12, respectively. In general, it is revealed from Figs 11 and 12 that both VL’s and GAS’s fatigue were progressively developing during the cycling exercise. However, in comparison to CSI, the rate of increase in FPM seemed generally smaller (from 0.21 to 0.40). We may speculate that this could be partly attributed to that the subjects were continuously trying to adjust and change their postures and attitudes during the cycling motion so the progressive development of muscle fatigue may slow down [44].

### A multivariate linear regression analysis used to relate the Borg rating of perceived exertion (RPE) value to the FPM and CSI

In general, the main purpose of the linear regression analysis here is to use mathematical expressions to describe the Borg RPE scale in terms of the FPM and/or CSI. The scenario of our numerical experiment is described as follows. First, we let the Borg RPE be the dependent variable, and let both the FPM and CSI be independent variables. That is, we have

$$\text{Borg RPE}=C_{FPM\_VL}\cdot \text{FPM\_VL}+C_{FPM\_GAS}\cdot \text{FPM\_GAS}+C_{CSI}\cdot \text{CSI}+C_{0}$$ (5)

where *C*_*FPM_VL*_, *C*_*FPM_GAS*_, *C*_*CSI*_ and *C*_0_ are unknown constants called the modeling parameters. Note that the coefficients *C*_*FPM_VL*_, *C*_*FPM_GAS*_, *C*_*CSI*_ control the behavior of the model and can be used to quantify the attribute and strength of the relationship between Borg RPE scale and FPM of VL and GAS muscles, and CSI, respectively. Moreover, a quantity referred to as the coefficient of determination *R* ^2^, ranging from 0 to 1, is used to measure the proportion of the dependent variable, i.e., Borg scale, attributable to the information obtained from the independent variables, FPM_VL, FPM_GAS, and CSI, in the multivariate linear model.

As described previously, FPM of VL and GAS, as well as CSI were evaluated and updated as time evolved. Also, the Borg RPE scale value was synchronously recorded during the cycling test. Therefore, we first performed a multivariate linear regression analysis, as indicated in (5), on the Borg scale, FPM and CSI data derived from each subject and tabulated all the statistical analysis results in Table 1. It is observed from Table 1 that all the coefficients except *C*_*FPM_GAS*_ were positive, indicating that the FPM of VL muscle and CSI were positively correlated with the Borg value. It should be also noted that although the mean of *C*_*FPM_GAS*_ was negative, it may not be able to reach a satisfactory significance level (since *p* = 0.259). Moreover, the averaged coefficient of determination *R* ^2^ (*i*.*e*., computed over all the twenty-two subjects) was 0.86, indicating that 86% of the variation in the Borg RPE scale can be explained by a joint analysis of FPM and CSI in the linear model.

**Table 1:** Statistical analysis results of the modeling parameter estimation derived from the linear regression model in (3).

Independent variable	Coefficient	*p* value
FPM_VL	7.34 + 4.24	0.091
FPM_GAS	-0.09 + 2.71	0.259
CSI	8.01 + 1.77	0.094
*C*_0_ (intercept)	10.68 + 0.09	<0.01

Secondly, we related the Borg scale to the FPM data only using the linear regression model as follows:

$$\text{Borg RPE}=C_{FPM\_VL}\cdot \text{FPM\_VL}+C_{FPM\_GAS}\cdot \text{FPM\_GAS}+C_{0}$$ (6)

Similarly, we tabulated all the statistical results in Table 2. Observing Table 2, we may see that the mean values of coefficients *C*_*FPM_VL*_ and *C*_*FPM_GAS*_ were both positive, indicating that the FPM of VL and GAS muscles were both positively correlated with the Borg value, as expected. Also, the mean value of *R* ^2^ was only 0.68, indicating that only 68% of the variation in the Borg RPE scale can be explained by FPM of VL and GAS in the linear model.

**Table 2:** Statistical analysis results of the modeling parameter estimation derived from the linear regression model in (4).

Independent variable	Coefficient	*p* value
FPM_VL	4.62 + 5.90	0.079
FPM_GAS	4.05 + 3.19	0.031
*C*_0_ (intercept)	13.37 + 0.85	0.039

Finally, we quantified the association between the Borg scale level and the cardiac stress by fitting the Borg scale and CSI data to the following linear regression model

$$\text{Borg RPE}=C_{CSI}\cdot \text{CSI}+C_{0}$$ (7)

and then tabulated all the statistical results in Table 3. Observing Table 3, again we see that the mean value of coefficient *C*_*CSI*_ was positive with *p*<0.01 (*p* = 0.0007), suggesting that the CSI was significantly positively correlated with the Borg RPE value. In addition, the mean value of *R* ^2^ was also 0.68 in this case, evidently indicating that there was 68% of the variation in the Borg RPE scale, at most, attributable to the non-motor features of fatigue due to cardiac stress in the linear model.

**Table 3:** Statistical analysis results of the modeling parameter estimation derived from the linear regression model in (5).

Independent variable	Coefficient	*p* value
CSI	11.00 + 1.21	< 0.01
*C*_0_ (intercept)	10.23 + 0.66	< 0.01

## Discussion

First, it should be noted that analysis results produced by numerical experiments as presented above have substantially validated the use of CSI in cardiac stress quantification. Secondly, just as the FPM, a typical CSI curve also shows an exponential-like increase as time evolves after the time instant at which the cardiac stress starts to increase (or the cardiac endurance starts to decrease). Further observing and comparing results presented in Fig 8 and Figs 11 and 12, one may find that CSI seemed to increase more quickly than did the FPM. As mentioned previously, this could be partly attributed to that subjects might adaptively and spontaneously regulate muscle groups for reducing local muscle fatigue by adjusting their postures and attitudes. As a result, this could effectively defer the progressive fatigue of a specific muscle such as VL or GAS, thus reducing the increase rate of FPM.

Moreover, it has been widely accepted that the Borg RPE scale can be used to rate the degree of perceived intensity of exercise [36]. In our experiments, participants were also asked to rate their perception of the exertion to express how they felt for the exercise intensity. We speculate that this RPE value, to a certain extent, may not only reflect the physical fatigue, but also reflect the cardiac stress status. Therefore, a multivariate linear regression analysis was performed to further evaluate how the Borg RPE value was correlated with the FPM and/or the CSI. In this aspect, we experimentally studied the performances of linear regression on three models formed by relating the Borg scale to FPM and CSI, to FPM only, and to CSI only, as indicated in (5), (6), and (7), respectively. First, according to the results as provided in Tables 1–3 one may see that while both the models in (6) and (7) achieved 68% *R* ^2^ value, the model in (5) achieved 86%, indicating that the Borg scale may be attributable to both the local muscle fatigue and cardiac stress. Secondly, considering the model in (5), we may see from Table 1 that while the Borg RPE scale generally increased only about 0.734 on scale for 0.1 added to FPM of VL muscles, it increased approximately 0.801 on scale for 0.1 added to CSI, implying that the effect on Borg scale due to CSI might be slightly dominant over that due to FPM of VL. On the other hand, the effect due to FPM of GAS might be negligible in this model. In addition, we also see from the numerical results in Table 1 that the mean intercept *C*_0_ = 10.68 actually implied that either the muscle fatigue or cardiac stress might start to contribute to Borg scale increment from Borg = 11.

Furthermore, consider the case of relating the Borg scale to FPM only, as indicated by the linear model in (6). First, we may see from the analysis results as provided in Table 2 that the averaged coefficient of determination *R* ^2^ was only 68%, manifesting that in addition to local muscle fatigue, the Borg scale should be also attributable to the factors other than muscle fatigue. Secondly, we found that the effects on Borg scale due to FPMs of VL and GAS muscles, respectively, were almost comparable. Thirdly, another interesting note is in this case the mean intercept *C*_0_ was 13.37, which indicated that the muscle fatigue might start to contribute to Borg scale increment from Borg = 13 (i.e., indicating that the exercise intensity is “somewhat hard”) or so. That is, this might imply that the onset of muscle fatigue generally occurred when subjects rated 13 on Borg scale. It is worth noting that such an observation is actually consistent to that found in our previous work [26].

Similarly, we also attempted to relate the Borg scale solely to CSI using the linear model as expressed in (7) and obtained the results as shown in Table 3. It is revealed from Table 3 that although the averaged coefficient of determination *R* ^2^ was only 68%, both the statistical results of *C*_*CSI*_ and *C*_0_ achieved the highest significant level (with *p*<0.01), in comparison to the numerical results as tabulated in Tables 1 and 2. According to the results as shown in Table 3, we may see that, as mentioned previously, CSI was positively correlated with the Borg RPE scale. In addition, we also found that here the mean intercept *C*_0_ was 10.23, which was actually consistent to the results in Table 1. That is, this number simply indicated that the cardiac stress might generally start to contribute to Borg scale increment since Borg = 11. In fact, according to the Borg RPE scale subjects may rate their perception of the exertion 11 on scale when they feel the exercise intensity is “fairly light” and the effort level corresponding to this value is 60% of maximum effort [36]. Therefore, we may further conclude that such an exercise intensity might be thought of as being a possible threshold for substantially, effectively initiating the cardiac stress “overload,” and also, the onset of cardiac stress elevation seemed to generally occur prior to the onset of muscle fatigue.

In summary, the numerical experimental results produced by a nonlinear analysis of HRV indicated that the cycling exercise effects on HR dynamics can be continuously and quantitatively captured by CSI. Moreover, we further incorporated the CSI into our system so the onset of the occurrence of non-motor features of fatigue due to cardiac stress “overload” can be explicitly determined from the CSI tracings. In the authors’ opinion, in addition to providing an on-line muscle fatigue monitoring and analysis, the proposed system may be also used in sports medicine, or used by cardiologists who carried out stress tests for monitoring heart condition in patients with heart diseases, thus representing a significant benefit from this study.

## Conclusion

In this paper, a cycling-based training system for real-time muscle fatigue and cardiac stress monitoring and analysis is introduced. In fact, RPE scale has been widely accepted as a convenient index which is often used to determine the level of fatigue during physical exercise. However, since fatigue induced by exercise is a complex phenomenon which may not be solely related to RPE, our study thus aimed to develop novel indices, other than RPE, and seek for their applicability for objectively and quantitatively monitoring and assessing muscle fatigue and cardiac stress during physical exercise. A new parameter, dubbed the CSI, used to quantify the cardiac stress status of subjects undergoing cycling exercise was developed. Similar to how the FPM was used to monitor the progression of muscle fatigue, the CSI was also incorporated into the system that was developed in our previous work for continuously and quantitatively assessing the progression of cardiac stress during exercise. In addition, in order to validate the applicability of FPM and CSI into exercise-induced fatigue analysis, we further related the Borg RPE to both the FPM and CSI using a multivariate linear regression analysis. Numerical experimental results indicated that the Borg RPE was generally positively correlated with FPM and/or CSI, suggesting that the motor factors and the factors due to cardiac stress might both contribute to fatigue state during physical exercise.
